# Risk Factors for Severe Maternal Morbidity Among Women Enrolled in Mississippi Medicaid

**DOI:** 10.1001/jamanetworkopen.2023.50750

**Published:** 2024-01-08

**Authors:** Shishir Maharjan, Swarnali Goswami, Yiran Rong, Terri Kirby, Dennis Smith, Catherine X. Brett, Eric L. Pittman, Kaustuv Bhattacharya

**Affiliations:** 1 Department of Pharmacy Administration, University of Mississippi School of Pharmacy, University; 2 Center for Pharmaceutical Marketing and Management, University of Mississippi School of Pharmacy, University; 3 Now with Complete Health Economics and Outcomes Solutions, LLC, Chalfont, Pennsylvania; 4 Now with MedTech Epidemiology and Real-World Data Sciences, Johnson and Johnson, New Brunswick, New Jersey; 5 Mississippi Division of Medicaid, Jackson; 6 Alliant Health Solutions, Jackson, Mississippi

## Abstract

**Importance:**

Mississippi has one of the highest rates of severe maternal morbidity (SMM) in the US, and SMMs have been reported to be more frequent among Medicaid-insured women. A substantial proportion of pregnant women in Mississippi are covered by Medicaid; hence, there is a need to identify potential risk factors for SMM in this population.

**Objective:**

To examine the associations of health care access and clinical and sociodemographic characteristics with SMM events among Mississippi Medicaid–enrolled women who had a live birth.

**Design, Setting, and Participants:**

A nested case-control study was conducted using 2018 to 2021 Mississippi Medicaid administrative claims database. The study included Medicaid beneficiaries aged 12 to 55 years who had a live birth and were continuously enrolled throughout their pregnancy period and 12 months after delivery. Individuals in the case group had SMM events and were matched to controls on their delivery date using incidence density sampling. Data analysis was performed from June to September 2022.

**Exposure:**

Risk factors examined in the study included sociodemographic factors (age and race), health care access (distance from delivery center, social vulnerability index, and level of maternity care), and clinical factors (maternal comorbidity index, first-trimester pregnancy-related visits, and postpartum care).

**Main Outcomes and Measures:**

The main outcome of the study was an SMM event. Adjusted odds ratio (aORs) and 95% CIs were calculated using conditional logistic regression.

**Results:**

Among 13 485 Mississippi Medicaid–enrolled women (mean [SD] age, 25.0 [5.6] years; 8601 [63.8%] Black; 4419 [32.8%] White; 465 [3.4%] other race [American Indian, Asian, Hispanic, multiracial, and unknown]) who had a live birth, 410 (3.0%) were in the case group (mean [SD] age, 26.8 [6.4] years; 289 [70.5%] Black; 112 [27.3%] White; 9 [2.2%] other race) and 820 were in the matched control group (mean [SD] age, 24.9 [5.7] years; 518 [63.2%] Black; 282 [34.4%] White; 20 [2.4%] other race). Black individuals (aOR, 1.44; 95% CI, 1.08-1.93) and those with higher maternal comorbidity index (aOR, 1.27; 95% CI, 1.16-1.40) had higher odds of experiencing SMM compared with White individuals and those with lower maternal comorbidity index, respectively. Likewise, an increase of 100 miles (160 km) in distance between beneficiaries’ residence to the delivery center was associated with higher odds of experiencing SMM (aOR, 1.14; 95% CI, 1.07-1.20).

**Conclusions and Relevance:**

The study findings hold substantial implications for identifying high-risk individuals within Medicaid programs and call for the development of targeted multicomponent, multilevel interventions for improving maternal health outcomes in this highly vulnerable population.

## Key Points

**Question:**

What are the different risk factors associated with severe maternal morbidity (SMM) among women with a live birth enrolled in Medicaid?

**Findings:**

In this nested case-control study of 13 485 women with live births enrolled in Mississippi Medicaid from 2018 to 2021, 410 beneficiaries (3.0%) had any SMM. Race, maternal comorbidity index, and distance of beneficiary’s residence from the delivery center were associated with a significantly higher risk of SMM.

**Meaning:**

The findings of this study may help identify women enrolled in Medicaid programs who are at the highest risk for SMM and aid in developing targeted multicomponent, multilevel interventions for improving maternal health outcomes in this highly vulnerable population.

## Teaser

This case-control study examines the association of health care access and clinical and sociodemographic characteristics with severe maternal morbidity events among Mississippi Medicaid–enrolled women who had a live birth.

## Introduction

Poor maternal health is a major public health concern in the US, with the country having a higher maternal mortality rate than any other developed nation.^1,2^ Despite substantial investments in technology and services aimed at improving maternal health, the remarkably high maternal mortality rate underscores the necessity for comprehensive research focusing on maternal morbidities and the corresponding risk factors. The southern region of the US,^3^ especially Mississippi, is greatly affected by maternal health issues.^4^ Compared with the national average of 17.4 cases per 100 000 live births, Mississippi has one of the highest rates of maternal mortality in the country (22.1 cases per 100 000 live births).^4^

According to the Centers for Disease Control and Prevention (CDC), severe maternal morbidity (SMM) is defined as “unexpected outcomes of labor and delivery that result in significant short- or long-term consequences to a woman’s health.”^5^ It encompasses a spectrum of severe complications following pregnancy and childbirth, including such conditions as eclampsia, acute renal failure, cardiac arrest, and others, all of which have detrimental impact on a woman’s health. In recent years, SMM has become an important marker for examining disparities in maternal health outcomes.^6^ The annual rate of SMM in the US has seen a great increase in recent decades, doubling from 49.5 per 10 000 births in 1993 to 144 per 10 000 births in 2014.^7^ Despite the comprehensive recommendations provided by the CDC and the American College of Obstetricians and Gynecologists for monitoring and assessing severe pregnancy and delivery complications,^7,8,9^ the estimated number of SMM cases surpasses 60 000 annually.^1^ More than 80% of pregnancy-related deaths in the US could have been prevented, yet inadequate treatment and the failure to identify health risk factors contribute to hundreds of maternal deaths annually.^10^ In the literature, a number of risk factors for SMM and mortality have been identified, including maternal age, cesarean delivery, multifetal gestation, obesity, and preexisting chronic conditions.^11,12,13,14,15,16,17,18,19^ There is also evidence that additional factors, including obstetric and medical factors, unmarried status, low maternal education, and rural residency, may potentially increase the risk of SMM and pregnancy-related mortality.^18,19,20,21,22,23,24,25^ Although these clinical and sociodemographic factors are critical in understanding the maternal health crisis, it is equally important to investigate factors associated with availability, quality, and accessibility of maternity care resources.^26^ These health care access factors will help to address spatial disparities and resource allocation in maternal care access in a particular region. This demands the careful investigation of the influence of understudied clinical factors and health care access parameters in maternal health outcomes, particularly in Medicaid-enrolled populations.

Among 26 states that have reported SMM data, Mississippi has the highest rate of SMM.^4^ The Medicaid program serves as the primary coverage source for maternal care in Mississippi, with more than 60% of pregnant women in the state relying on Medicaid for their health care needs.^4^ A study^27^ has demonstrated that Medicaid-insured women have a greater risk of experiencing SMM than their commercially insured counterparts. Given that SMM has been shown to occur more frequently among Medicaid-insured women, there is a critical need to investigate the factors contributing to the mounting concerns in this population. Therefore, the goal of this study is to assess the association of health care access and clinical and sociodemographic characteristics with SMM among women who had a live birth and were enrolled in Mississippi Medicaid.

## Methods

### Study Design and Data Source

This nested case-control study used deidentified 2018 to 2021 Mississippi Medicaid coordinated care organization and fee-for-service administrative claims data. The study was approved by the University of Mississippi institutional review board with a waiver of informed consent because of the retrospective nature of the data, in accordance with 45 CFR §46. The study followed the Strengthening the Reporting of Observational Studies in Epidemiology (STROBE) reporting guidelines.^28^

### Study Cohort Definition

Beneficiaries enrolled in Mississippi Medicaid who had a live birth between January 1, 2018, and December 31, 2020, were identified using the *International Statistical Classification of Diseases, Tenth Revision, Clinical Modification (ICD-10-CM)* codes for live birth (eTable 1 in Supplement 1) following the approach used by Moll et al.^29^ The cohort entry date or delivery date was defined according to the date of the first claim indicating live birth during delivery hospitalization. Preterm or full-term status of the delivery was identified using *ICD-10-CM* codes (eTable 1 in Supplement 1). The pregnancy start date was estimated on the basis of the algorithm previously used by Moll et al.^29^ Beneficiaries who were not continuously enrolled during the pregnancy period and 12 months after cohort entry date, who were younger than 12 years or older than 55 years at cohort entry date, and who were transferred to another institution were omitted from the study.

### Case and Control Definitions

Medicaid beneficiaries who experienced any SMM events during 12 months after cohort entry date were classified as cases. An SMM event was determined as 1 of the 21 conditions defined by the CDC for identification of SMM^30^ (eTable 2 in Supplement 1). The index date was defined as the date of the first occurrence of any SMM event (Figure). Controls were defined as individuals from study cohort who had not experienced SMM from the delivery date until the point or time they were matched with their respective cases. The matching was performed in a way that ensured that controls had not experienced any SMM events up to the time of their respective matched cases. On the basis of the delivery date, cases and controls were matched in a 1:2 ratio, using incidence density sampling.^31^ This technique allowed for controls to be randomly sampled from individuals included in the study sample, such that each control had similar or longer time at risk for SMM as the corresponding matched case. The index date for controls was assigned as of their matched cases’ index date.

**Figure.**
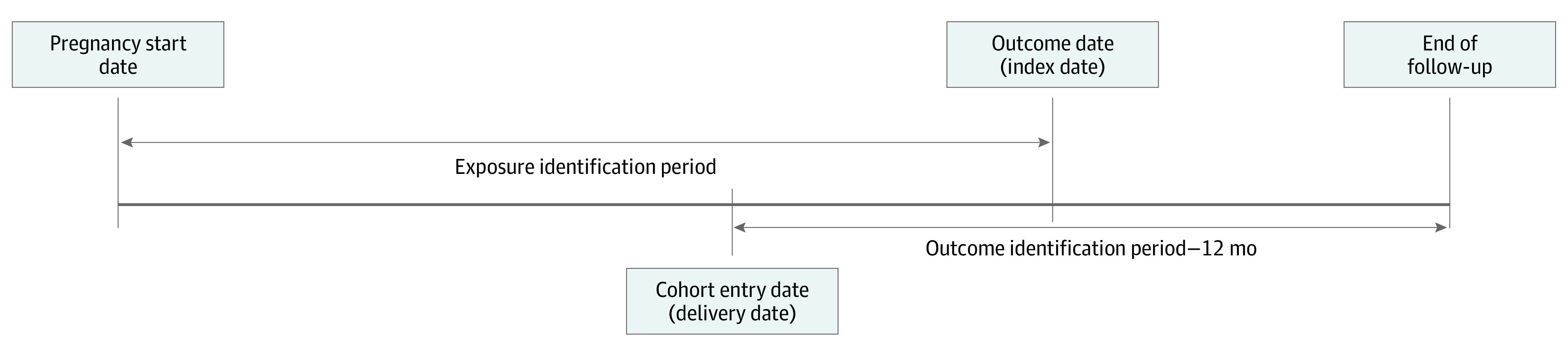
Methodological Diagram of the Study

### Independent Variables

#### Sociodemographic Characteristics

The sociodemographic factors included age and race (classified as Black, White, and other, which includes American Indian, Asian, Hispanic, multiracial, and unknown). Information on race and ethnicity were extracted from the Mississippi Medicaid beneficiary enrollment and demographic summary file. Because of sample size limitations of this study, race categories were limited to Black and White, with the remaining race categories grouped into other. Race is an important social construct and was included in the study analysis on the basis of prior evidence of racial disparities in maternal health outcomes. The age of individuals was calculated as of their delivery date.

#### Health Care Access

Health care access was assessed using the social vulnerability index (SVI), distance of the beneficiaries’ residence from the delivery center, and the level of maternity care. SVI is a neighborhood-based measure that is calculated according to the county of the beneficiary’s residence. It was categorized as least vulnerable (below first quartile), moderately vulnerable (between first and third quartile), and most vulnerable (above third quartile).^32^ Similarly, the distance that beneficiaries had to travel for the delivery was determined at the zip code level according to the beneficiary’s residence and the location of the delivery center. Level of maternity care was categorized as access to maternity care, low access to care (few delivery centers, obstetric practitioners, or a high proportion of women without health insurance), and maternity care desert (limited or entirely absent maternal health care services).^33^

#### Clinical Characteristics

The maternal comorbidity index (MCI), which was developed and validated by Bateman et al,^34^ was measured for each eligible beneficiary to capture the burden of chronic, behavior, and pregnancy-induced conditions (eTable 3 in Supplement 1).^35^ The conditions underlying MCI were assessed from pregnancy start date to index date. Additional clinical characteristics included first-trimester pregnancy-related visits and postpartum care visits in the 2 weeks after delivery.

### Statistical Analysis

Data analysis was performed from June to September 2022. The health care access, sociodemographic, and clinical characteristics of the study cohort were summarized using descriptive statistics. For categorical variables, frequency and percentage distributions were reported. McNemar test or Cochran-Mantel-Haenszel test was used to test statistical differences between cases and controls. Continuous variables were presented using mean (SD), and paired *t* tests were used for testing statistically significant differences between the cases and controls. The association between independent variables and SMM was tested using conditional logistic regression. All health care access, sociodemographic, and clinical factors were included in the adjusted model. For all statistical analyses, 2-sided tests with α = .05 were used for significance. SAS statistical software version 9.4 (SAS Institute) was used for data management and statistical analyses.

## Results

From 2018 to 2020, a total of 43 599 Medicaid beneficiaries had a live birth. Among them, 30 114 beneficiaries could not satisfy the continuous eligibility requirement or age criteria (aged 12-55 years as of the delivery date), were transferred to another institution, or had missing information and, hence, were excluded from the study. Finally, there were 13 485 beneficiaries with live birth who were eligible for the study.

### Characteristics of Eligible Beneficiaries

Table 1 presents the health care access, clinical, and sociodemographic characteristics of the eligible study cohort. Most of the beneficiaries were Black (8601 beneficiaries [63.8%]) and aged 18 to 34 years (11 730 beneficiaries [87.0%]), with the mean (SD) age being 25.0 (5.6) years; 4419 beneficiaries (32.8%) were White and 465 (3.4%) were other race. According to the SVI, more than one-half of the study cohort belonged to the moderately vulnerable group (7402 beneficiaries [55.0%]), 8852 beneficiaries (65.6%) had access to maternity care, and the mean (SD) distance traveled by beneficiaries for delivery was determined to be 114.7 (226.8) miles (183.5 [362.8] km). The median (IQR) distance traveled by beneficiaries for delivery was 34.2 (9.4-102.4) miles (54.7 [15.0-163.8] km). With respect to clinical factors, 52.1% of the study cohort (7020 beneficiaries) had pregnancy-related visits during the first trimester, and 30.0% (4045 beneficiaries) had postpartum care visits in the 2 weeks following delivery.

**Table 1.** Health Care Access, Sociodemographic, and Clinical Characteristics of the Eligible Cohort

Characteristic	Beneficiaries, No. (%)			*P* value
	Full cohort (N = 13 485)	Case (n = 410)	Control (n = 820)	
Age, mean (SD), y^a^	25.0 (5.6)	26.8 (6.4)	24.9 (5.7)	<.001
Age range, y				
<18	892 (6.6)	24 (5.8)	56 (6.8)	
18-34	11 730 (87.0)	333 (81.2)	705 (86.0)	.005
≥35	863 (6.4)	53 (12.9)	59 (7.2)	
Race^a^				
Black	8601 (63.8)	289 (70.5)	518 (63.2)	.04
White	4419 (32.8)	112 (27.3)	282 (34.4)	
Other^b^	465 (3.4)	9 (2.2)	20 (2.4)	
Distance from delivery center, miles^c^				
Mean (SD)	114.7 (226.8)	183.2 (336.3)	96.7 (191.2)	<.001
Median (IQR)	34.2 (9.4-102.4)	52.8 (14.2-188.6)	30.8 (9.3-91.3)	
Social vulnerability index^a^				
Least vulnerable	3382 (25.1)	104 (25.4)	206 (25.1)	.78
Moderately vulnerable	7402 (55.0)	225 (54.9)	437 (53.4)	
Most vulnerable	2680 (19.9)	81 (19.8)	176 (21.5)	
Pregnancy-related visits^d^	7020 (52.1)	256 (62.4)	377 (54.0)	.005
Postpartum care visits^e^	4045 (30.0)	122 (29.8)	236 (28.8)	.72
Level of maternity care^a^				
Access to maternity care	8852 (65.6)	273 (66.6)	515 (62.8)	.37
Low access to care	1334 (9.9)	32 (7.8)	78 (9.5)	
Maternity care desert	3299 (24.5)	105 (25.6)	227 (27.7)	
Maternal comorbidity index^f^	Not applicable	1.12 (1.65)	0.54 (1.14)	<.001

SI conversion factor: To convert miles to kilometers, multiply by 1.6.

^a^ Measured from the cohort entry date.

^b^ Other race includes American Indian, Asian, Hispanic, multiracial, and unknown.

^c^ Measured from the delivery date.

^d^ Measured from the first trimester of pregnancy.

^e^ Measured from 2 weeks after the delivery date.

^f^ Measured from the pregnancy start date to index date.

### Case and Controls

As shown in Table 2, 410 of the beneficiaries (3.0%) (mean [SD] age, 26.8 [6.4] years) in the eligible cohort experienced any SMM event. Among the SMM conditions, pulmonary edema and acute heart failure (92 beneficiaries [22.4%]) was the most common, followed by sepsis (90 beneficiaries [21.9%]) and adult respiratory distress syndrome (56 beneficiaries [13.7%]). A large proportion of the cases were aged 18 to 34 years (333 beneficiaries [81.2%]), were Black (289 beneficiaries [70.5%]), had access to maternity care (273 beneficiaries [66.6%]), and had moderate social vulnerability (225 beneficiaries [54.9%]); 112 cases (27.3%) were White and 9 (2.2%) were other race. Although more than one-half of cases (206 beneficiaries [50.2%]) experienced an SMM event within the first 6 weeks after delivery, 42.7% (175 beneficiaries) experienced their first SMM event after 12 weeks after delivery. The remaining 7.1% of the cases (29 beneficiaries) experienced an SMM event between 6 to 12 weeks after delivery. Moreover, 256 cases (62.4%) had pregnancy-related visits in the first trimester of pregnancy, and 122 cases (29.8%) had postpartum care visits. The mean (SD) MCI for cases was 1.12 (1.65) (median [IQR], 0 [0-2]). The mean (SD) distance from cases’ residence to the delivery center was 183.2 (336.3) miles (293.12 [538.14] km; median [IQR], 52.8 (14.2-188.6) miles; 84.5 [22.7-301.8] km) (Table 1).

**Table 2.** Severe Maternal Morbidity Conditions Among Beneficiaries Enrolled in Mississippi Medicaid With Live Births

Severe maternal morbidity conditions	Beneficiaries, No. (%) (N = 410)
Acute myocardial infarction	9 (2.2)
Aneurysm	2 (0.5)
Acute renal failure	44 (10.7)
Adult respiratory distress syndrome	56 (13.7)
Amniotic fluid embolism	6 (1.5)
Cardiac arrest and/or ventricular fibrillation	4 (1.0)
Conversion of cardiac rhythm	0
Disseminated intravascular coagulation	26 (6.3)
Eclampsia	48 (11.7)
Heart failure and/or arrest during surgery or procedure	0
Puerperal cerebrovascular disorders	51 (12.4)
Pulmonary edema and acute heart failure	92 (22.4)
Severe anesthesia complications	0
Sepsis	90 (21.9)
Shock	24 (5.8)
Sickle cell disease with crisis	13 (3.2)
Air and thrombotic embolism	45 (11.0)
Blood products transfusion	0
Hysterectomy	0
Temporary tracheostomy	0
Ventilation	0

The majority of the controls (820 beneficiaries [86.0%]) were aged 18 to 34 years (mean [SD] age, 24.9 [5.7] years), were Black (518 beneficiaries [63.2%]), had access to maternity care (515 beneficiaries [62.8%]), and had moderate social vulnerability (437 beneficiaries [53.4%]); 282 controls (34.4%) were White, and 20 (2.4%) were other race. In addition, 54.0% of the controls (377 beneficiaries) had first-trimester pregnancy-related visits, and 28.8% of the controls (236 beneficiaries) had postpartum care visits. The mean (SD) MCI for controls was 0.54 (1.14) (median [IQR], 0 [0-0]). The mean (SD) distance from controls’ residence to the delivery center was 96.7 (191.2) miles (154.7 [305.9] km). The median (IQR) distance traveled by controls for delivery was 30.8 (9.3-91.3) miles (49.3 [14.9-146.1] km). As shown in Table 1, cases and controls were significantly different in terms of race, age, distance from the delivery center, first-trimester pregnancy-related visits, and MCI. In addition, the unadjusted results of the potential risk factors are included in eTable 4 in Supplement 1.

### Adjusted Analysis

Table 3 displays the findings of the adjusted conditional logistic regression. After accounting for other variables, the odds of experiencing SMM increased by 27% for a single-point increase in MCI (adjusted odds ratio [aOR], 1.27; 95% CI, 1.16-1.40). In addition, every 100-mile (160-km) increase in the distance of the beneficiary’s residence from the delivery center was associated with a 14% increase in the odds of experiencing SMM (aOR, 1.14; 95% CI, 1.07-1.20). Moreover, beneficiaries aged 35 years or older had higher odds of experiencing SMM compared with beneficiaries aged 18 to 34 years (aOR, 1.49; 95% CI, 0.98-2.26), but the difference was not statistically significant. Furthermore, Black beneficiaries had a 44% greater odds of experiencing SMM (aOR, 1.44; 95% CI, 1.08-1.93) than White beneficiaries. No statistically significant associations were observed between other independent variables and SMM.

**Table 3.** Adjusted Associations of Risk Factors With Severe Maternal Morbidity

Characteristics	Adjusted OR (95% CI)	*P* value
Maternal comorbidity index	1.27 (1.16-1.40)	<.001
Distance from delivery center	1.14 (1.07-1.20)	<.001
Age group, y		
<18	1.01 (0.60-1.68)	.97
18-34	1 [Reference]	NA
≥35	1.49 (0.98-2.26)	.06
Race		
Black	1.44 (1.08-1.93)	.01
White	1 [Reference]	NA
Other^a^	1.05 (0.44-2.50)	.91
Pregnancy-related visit	1.14 (0.87-1.50)	.33
Postpartum care visit	1.12 (0.85-1.47)	.44
Social vulnerability index		
Least vulnerable	1 [Reference]	NA
Moderately vulnerable	0.89 (0.66-1.21)	.47
Most vulnerable	0.71 (0.47-1.08)	.11
Level of maternity care		
Access to maternity care	1 [Reference]	NA
Low access to care	0.87 (0.54-1.38)	.54
Maternity care desert	0.95 (0.70-1.28)	.72

Abbreviations: NA, not applicable; OR, odds ratio.

^a^ Other race includes American Indian, Asian, Hispanic, multiracial, and unknown.

## Discussion

This case-control study found that 3.0% of Mississippi Medicaid–enrolled women with a live birth experienced an SMM event, with the most common SMM events being pulmonary edema and acute heart failure, followed by sepsis. However, studies by Hirai et al^36^ and Fink et al^37^ found that intravascular coagulation and blood transfusion were the most common SMMs, respectively. The results of the current study also revealed that, in this population, there is a significant association of elevated risk of SMM with distance of the beneficiary’s residence to the delivery center, MCI score, and beneficiary’s race.

A higher MCI score has been associated with an increased risk of SMM, per extant literature. A study by Bateman et al,^34^ where MCI was initially created and validated in a Medicaid population, found that the likelihood of organ damage or death increased by 37% for every unit increase in MCI score in the 30 days after delivery. Another study,^22^ conducted among pregnant women in Texas, found a significant association between higher MCI scores and SMM risk during delivery-related hospitalizations. Similar findings were reported by Main et al^38^ in their analysis of California’s delivery hospital discharge data. Our analysis found that Medicaid-enrolled beneficiaries with higher MCI scores are more likely to experience SMM in the year following delivery, which is consistent with previous studies. This implies that MCI scores can be used as an effective tool to identify women at high risk of SMM, aiding implementation of tailored clinical care programs for avoiding such adverse outcomes.

In addition, our study showed that Black women had 44% greater odds of SMM than White women. This is in line with a study by Chen et al,^39^ which also reported higher odds for SMM among Medicaid-insured Black women compared with their White counterparts. Differences in broader socioeconomic factors and structural and social discrimination might be underlying the worse complications for Black women. Several health advocacy groups and organizations like the Society for Maternal Fetal Medicine and American College of Obstetricians and Gynecologists are committed to expanding efforts to mitigate such maternal disparities.^26^ Such initiatives and efforts should be prioritized in the states with high rates of maternal mortality and morbidity. Our study found that the odds of SMM increase by 14% for a 100-mile increase in the distance between a beneficiary’s residence and the delivery center. There is a dearth of literature on how distance from health care centers affects maternal health outcomes. A recent systematic literature review^40^ noted the disparity in the results from studies that assessed the association of traveling further for health services with health outcomes. As echoed by the systematic literature review, as well as the results of our study, the association of distance from delivery center with health outcomes, especially maternal outcomes, warrants further assessment in future studies.

Similarly, the study findings suggested that women aged 35 years and older had higher risk of SMM compared with those aged 18 to 34 years, although the difference was not statistically significant. This aligns with findings from Lisonkova et al,^41^ who found a marked increase in SMM with maternal age, particularly in women aged 35 years or older. A population-based retrospective cohort study^42^ conducted using birth certificate records from 2012 to 2016 supports this evidence. It found that individuals older than 40 years had the highest rates of SMM and that pregnancies at an advanced age carried a higher risk of SMM. Older women have a risk of cardiovascular, respiratory, and reproductive morbidity, which may manifest clinically and precipitate during pregnancy.^43,44,45^ In addition, level of maternity care was not found to be a significant factor in the study. However, the research sheds light on maternity care deserts, which are areas with limited access to maternal health care services. If left unaddressed, these maternal care deserts could exacerbate already existing inequalities in maternal health. Hence, policymakers, health care practitioners, and communities must address these gaps and ensure equitable access to high-quality maternal health care.^46^ The study did not find statistically significant results for first-trimester pregnancy-related visits and immediate postpartum visits within first 2 weeks. Future research should explore the impact of longer-term postpartum care on maternal outcomes. Nevertheless, the study underscores the importance of these services for maternal health and the need for further research and interventions to enhance the quality and accessibility of prenatal and postpartum care. The current study did not explicitly look at the impact of timing of SMM events on the association of risk factors of interest with SMM and, hence, should be examined in future research.

### Strengths and Limitations

Our study has several strengths. This study conducted an in-depth assessment of SMM by reporting the proportion of beneficiaries in the sample experiencing each of the 21 indicators of SMM. Second, this study also added to the current literature on SMM in the US by assessing pertinent factors such as MCI, SVI, pregnancy-related visits, postpartum care, level of maternity care, and distance between beneficiary’s residence and the delivery center. Third, the current study used a validated claims-based algorithm to identify delivery and pregnancy start date in claims data analyses.

This study is also subject to certain limitations. Only beneficiaries enrolled in Mississippi Medicaid with continuous enrollment were included in this study. Hence, caution should be exercised while extrapolating these findings to individuals other than Medicaid-enrolled women of childbearing age who had 12 months of postpartum coverage. Future studies should assess risk factors of SMM in other populations to confirm the findings reported in this study. Although our study used a validated claims-based algorithm to identify women with live births and to estimate pregnancy start date, errors or biases in the algorithm may affect the results. However, the algorithm has been commonly used in previous studies and has acceptable sensitivity and specificity.^47,48^ Furthermore, given the claim-based analysis, our study could not comprehensively account for all other potential confounders owing to unavailability and underrepresentation, such as body mass index, parity, and prenatal vitamin and aspirin use, and so forth. Future research, especially those using linked databases, should aim to investigate a broader set of confounding variables.

## Conclusions

In this case-control study, elevated risk of SMM was observed in Medicaid-enrolled women with live birth who had higher MCI scores, lived farther from the delivery center, or were Black. These findings have important implications for identifying high-risk individuals within Medicaid programs and developing targeted interventions that address multiple factors and levels to improve maternal health outcomes among this vulnerable population. Collaboration among policymakers, health care practitioners, and community leaders is crucial to implement interventions and programs aimed at reducing maternal morbidity and mortality. Maternal health care policies focusing on identifying women at risk of SMM and increasing access to high-quality, equitable maternity care should be prioritized in areas with high rates of maternal morbidity and mortality to mitigate disparities in maternal health.

## Notes

Supplement 1. **eTable 1.** *ICD-10* Codes for Pregnancy Characteristics **eTable 2.** Severe Maternal Morbidity Indicators **eTable 3.** Maternal Comorbidity Index **eTable 4.** Unadjusted Relationship Between Risk Factors and Severe Maternal Morbidity

Supplement 2.
Data Sharing Statement

## References

[1] Declercq E, Zephyrin LC. Severe maternal morbidity in the United States: a primer. October 28, 2021. Accessed December 1, 2023. https://www.commonwealthfund.org/publications/issue-briefs/2021/oct/severe-maternal-morbidity-united-states-primer

[2] Tikkanen R, Gunja MZ, FitzGerald M, Zephyrin L. Maternal mortality and maternity care US compared to 10 other developed countries. November 18, 2020. Accessed October 11, 2022. https://www.commonwealthfund.org/publications/issue-briefs/2020/nov/maternal-mortality-maternity-care-us-compared-10-countries

[3] Snyder JE, Stahl AL, Streeter RA, Washko MM. Regional variations in maternal mortality and health workforce availability in the United States. Ann Intern Med. 2020;173(11)(suppl):S45-S54. doi:10.7326/M19-3254 33253022

[4] Center for Mississippi Health Policy. Postpartum Medicaid: addressing gaps in coverage to improve maternal health. February 2021. Accessed October 11, 2022. https://mshealthpolicy.com/wp-content/uploads/2021/02/Post-Partum-Medicaid-Feb-2021.pdf

[5] Centers for Disease Control and Prevention. Severe maternal morbidity in the United States. February 2, 2021. Accessed October 11, 2022. https://www.cdc.gov/reproductivehealth/maternalinfanthealth/severematernalmorbidity.html

[6] Liese KL, Mogos M, Abboud S, Decocker K, Koch AR, Geller SE. Racial and ethnic disparities in severe maternal morbidity in the United States. J Racial Ethn Health Disparities. 2019;6(4):790-798. doi:10.1007/s40615-019-00577-w 30877505

[7] Thakkar A, Hameed AB, Makshood M, . Assessment and prediction of cardiovascular contributions to severe maternal morbidity. JACC Adv. 2023;2(2):100275. doi:10.1016/j.jacadv.2023.100275 37560021 PMC10410605

[8] Centers for Disease Control and Prevention. State strategies for preventing pregnancy-related deaths: a guide for moving maternal mortality review committee data to action. May 2022. Accessed October 11, 2022. https://www.cdc.gov/reproductivehealth/maternal-mortality/docs/pdf/State-Strategies-508.pdf

[9] American College of Obstetricians and Gynecologists. Severe maternal morbidity: screening and review. September 2016. Accessed October 11, 2022. https://www.acog.org/en/clinical/clinical-guidance/obstetric-care-consensus/articles/2016/09/severe-maternal-morbidity-screening-and-review

[10] Centers for Disease Control and Prevention. Pregnancy-related deaths: data from maternal mortality review committees in 36 US states, 2017-2019. September 26, 2022. Accessed October 24, 2022. https://www.cdc.gov/reproductivehealth/maternal-mortality/erase-mm/data-mmrc.html

[11] Osterman M, Hamilton B, Martin JA, Driscoll AK, Valenzuela CP. Births: final data for 2020. Natl Vital Stat Rep. 2021;70(17):1-50. 35157571

[12] Hinkle SN, Sharma AJ, Kim SY, . Prepregnancy obesity trends among low-income women, United States, 1999-2008. Matern Child Health J. 2012;16(7):1339-1348. doi:10.1007/s10995-011-0898-2 22009444

[13] Fisher SC, Kim SY, Sharma AJ, Rochat R, Morrow B. Is obesity still increasing among pregnant women? prepregnancy obesity trends in 20 states, 2003-2009. Prev Med. 2013;56(6):372-378. doi:10.1016/j.ypmed.2013.02.015 23454595 PMC4424789

[14] Campbell KH, Savitz D, Werner EF, . Maternal morbidity and risk of death at delivery hospitalization. Obstet Gynecol. 2013;122(3):627-633. doi:10.1097/AOG.0b013e3182a06f4e 23921870

[15] Small MJ, James AH, Kershaw T, Thames B, Gunatilake R, Brown H. Near-miss maternal mortality: cardiac dysfunction as the principal cause of obstetric intensive care unit admissions. Obstet Gynecol. 2012;119(2 pt 1):250-255. doi:10.1097/AOG.0b013e31824265c7 22270275

[16] Barber EL, Lundsberg LS, Belanger K, Pettker CM, Funai EF, Illuzzi JL. Indications contributing to the increasing cesarean delivery rate. Obstet Gynecol. 2011;118(1):29-38. doi:10.1097/AOG.0b013e31821e5f65 21646928 PMC3751192

[17] Callaghan WM, Creanga AA, Kuklina EV. Severe maternal morbidity among delivery and postpartum hospitalizations in the United States. Obstet Gynecol. 2012;120(5):1029-1036. doi:10.1097/AOG.0b013e31826d60c5 23090519

[18] Singh GK. Trends and social inequalities in maternal mortality in the United States, 1969-2018. Int J MCH AIDS. 2021;10(1):29-42. doi:10.21106/ijma.444 33442490 PMC7792749

[19] Black CM, Vesco KK, Mehta V, Ohman-Strickland P, Demissie K, Schneider D. Incidence of severe maternal morbidity during delivery hospitalization in U.S. commercially insured and Medicaid populations. J Womens Health (Larchmt). 2022;31(1):91-99. doi:10.1089/jwh.2020.8556 33891488 PMC11896018

[20] Nelson DB, Moniz MH, Davis MM. Population-level factors associated with maternal mortality in the United States, 1997-2012. BMC Public Health. 2018;18(1):1007. doi:10.1186/s12889-018-5935-2 30103716 PMC6090644

[21] Nik Hazlina NH, Norhayati MN, Shaiful Bahari I, Mohamed Kamil HR. The prevalence and risk factors for severe maternal morbidities: a systematic review and meta-analysis. Front Med (Lausanne). 2022;9:861028. doi:10.3389/fmed.2022.861028 35372381 PMC8968119

[22] Salahuddin M, Mandell DJ, Lakey DL, . Maternal comorbidity index and severe maternal morbidity during delivery hospitalizations in Texas, 2011-2014. Birth. 2020;47(1):89-97. doi:10.1111/birt.12465 31659788

[23] Lazariu V, Nguyen T, McNutt LA, Jeffrey J, Kacica M. Severe maternal morbidity: a population-based study of an expanded measure and associated factors. PLoS One. 2017;12(8):e0182343. doi:10.1371/journal.pone.0182343 28787028 PMC5546569

[24] Howland RE, Angley M, Won SH, . Determinants of severe maternal morbidity and its racial/ethnic disparities in New York city, 2008-2012. Matern Child Health J. 2019;23(3):346-355. doi:10.1007/s10995-018-2682-z 30712089

[25] Admon LK, Dalton VK, Kolenic GE, . Comparison of delivery-related, early and late postpartum severe maternal morbidity among individuals with commercial insurance in the US, 2016 to 2017. JAMA Netw Open. 2021;4(12):e2137716. doi:10.1001/jamanetworkopen.2021.37716 34878553 PMC8655600

[26] Howell EA. Reducing disparities in severe maternal morbidity and mortality. Clin Obstet Gynecol. 2018;61(2):387-399. doi:10.1097/GRF.0000000000000349 29346121 PMC5915910

[27] Interrante JD, Tuttle MS, Admon LK, Kozhimannil KB. Severe maternal morbidity and mortality risk at the intersection of rurality, race and ethnicity, and Medicaid. Womens Health Issues. 2022;32(6):540-549. doi:10.1016/j.whi.2022.05.003 35760662

[28] von Elm E, Altman DG, Egger M, Pocock SJ, Gøtzsche PC, Vandenbroucke JP; STROBE Initiative. The Strengthening the Reporting of Observational Studies in Epidemiology (STROBE) statement: guidelines for reporting observational studies. Lancet. 2007;370(9596):1453-1457. doi:10.1016/S0140-6736(07)61602-X 18064739

[29] Moll K, Wong HL, Fingar K, . Validating claims-based algorithms determining pregnancy outcomes and gestational age using a linked claims-electronic medical record database. Drug Saf. 2021;44(11):1151-1164. doi:10.1007/s40264-021-01113-8 34591264 PMC8481319

[30] Centers for Disease Control and Prevention. How does CDC identify severe maternal morbidity? February 8, 2021. Accessed October 19, 2022. https://www.cdc.gov/reproductivehealth/maternalinfanthealth/smm/severe-morbidity-ICD.htm

[31] Richardson DB. An incidence density sampling program for nested case-control analyses. Occup Environ Med. 2004;61(12):e59. doi:10.1136/oem.2004.014472 15550597 PMC1740694

[32] Centers for Disease Control and Prevention; Agency for Toxic Substances and Disease Registry; Geospatial Research, Analysis, and Services Program. CDC/ATSDR social vulnerability index, 2018, database MS. August 27, 2021. Accessed October 19, 2022. https://www.atsdr.cdc.gov/placeandhealth/svi/data_documentation_download.html

[33] March of Dimes. 2022 March of Dimes report card for United States. Accessed May 8, 2023. https://www.marchofdimes.org/peristats/reports/united-states/report-card

[34] Bateman BT, Mhyre JM, Hernandez-Diaz S, . Development of a comorbidity index for use in obstetric patients. Obstet Gynecol. 2013;122(5):957-965. doi:10.1097/AOG.0b013e3182a603bb 24104771 PMC3829199

[35] Metcalfe A, Lix LM, Johnson JA, . Validation of an obstetric comorbidity index in an external population. BJOG. 2015;122(13):1748-1755. doi:10.1111/1471-0528.13254 25559311 PMC5006847

[36] Hirai AH, Owens PL, Reid LD, Vladutiu CJ, Main EK. Trends in severe maternal morbidity in the US across the transition to *ICD-10-CM/PCS* from 2012-2019. JAMA Netw Open. 2022;5(7):e2222966. doi:10.1001/jamanetworkopen.2022.22966 35900764 PMC9335134

[37] Fink DA, Kilday D, Cao Z, . Trends in maternal mortality and severe maternal morbidity during delivery-related hospitalizations in the United States, 2008 to 2021. JAMA Netw Open. 2023;6(6):e2317641. doi:10.1001/jamanetworkopen.2023.17641 37347486 PMC10288331

[38] Main EK, Leonard SA, Menard MK. Association of maternal comorbidity with severe maternal morbidity: a cohort study of California mothers delivering between 1997 and 2014. Ann Intern Med. 2020;173(11)(suppl):S11-S18. doi:10.7326/M19-3253 33253023

[39] Chen J, Cox S, Kuklina EV, Ferre C, Barfield W, Li R. Assessment of incidence and factors associated with severe maternal morbidity after delivery discharge among women in the US. JAMA Netw Open. 2021;4(2):e2036148. doi:10.1001/jamanetworkopen.2020.36148 33528553 PMC7856547

[40] Kelly C, Hulme C, Farragher T, Clarke G. Are differences in travel time or distance to healthcare for adults in global north countries associated with an impact on health outcomes? a systematic review. BMJ Open. 2016;6(11):e013059. doi:10.1136/bmjopen-2016-013059 27884848 PMC5178808

[41] Lisonkova S, Potts J, Muraca GM, . Maternal age and severe maternal morbidity: a population-based retrospective cohort study. PLoS Med. 2017;14(5):e1002307. doi:10.1371/journal.pmed.1002307 28558024 PMC5448726

[42] Carr RC, McKinney DN, Cherry AL, Defranco EA. Maternal age-specific drivers of severe maternal morbidity. Am J Obstet Gynecol MFM. 2022;4(2):100529. doi:10.1016/j.ajogmf.2021.100529 34798330

[43] American College of Obstetricians and Gynecologists. Female age-related fertility decline. March 2014. Accessed December 16, 2022. https://www.acog.org/en/clinical/clinical-guidance/committee-opinion/articles/2014/03/female-age-related-fertility-decline

[44] Lapinsky SE. Acute respiratory failure in pregnancy. Obstet Med. 2015;8(3):126-132. doi:10.1177/1753495X15589223 27512467 PMC4935019

[45] De Viti D, Malvasi A, Busardò F, Beck R, Zaami S, Marinelli E. Cardiovascular outcomes in advanced maternal age delivering women: clinical review and medico-legal issues. Medicina (Kaunas). 2019;55(10):658. doi:10.3390/medicina55100658 31569595 PMC6843194

[46] Sonenberg A, Mason DJ. Maternity care deserts in the US. JAMA Health Forum. 2023;4(1):e225541. doi:10.1001/jamahealthforum.2022.5541 36633853

[47] Meinhofer A, Martinez ML, Palmsten K. Patterns in prescription opioids, benzodiazepines, and stimulants filled by pregnant Medicaid beneficiaries. JAMA Pediatr. 2023;177(2):210-213. doi:10.1001/jamapediatrics.2022.4892 36574236 PMC9857806

[48] Davies HG, Bowman C, Watson G, . Standardizing case definitions for monitoring the safety of maternal vaccines globally: GAIA definitions, a review of progress to date. Int J Gynaecol Obstet. 2023;162(1):29-38. doi:10.1002/ijgo.14843 37194339

