# Supplementary material for: Risk Factors for Severe Maternal Morbidity Among Women Enrolled in Mississippi Medicaid
Source: JAMA Netw Open. 2024 Jan 8;7(1):e2350750. doi: 10.1001/jamanetworkopen.2023.50750 (PMC10774990; doi:10.1001/jamanetworkopen.2023.50750)
Supplement: Supplement 1. — eTable 1. ICD-10 Codes for Pregnancy Characteristics eTable 2. Severe Maternal Morbidity Indicators eTable 3. Maternal Comorbidity Index eTable 4. Unadjusted Relationship Between Risk Factors and Severe Maternal Morbidity [file jamanetwopen-e2350750-s001.pdf]

Supplemental Online Content

Maharjan S, Goswami S, Rong Y, et al. Risk factors for severe maternal morbidity among women enrolled in Mississippi Medicaid. *JAMA Netw Open*. 2024;7(1):e2350750. doi:10.1001/jamanetworkopen.2023.50750

eTable 1. *ICD-10* Codes for Pregnancy Characteristics

eTable 2. Severe Maternal Morbidity Indicators

eTable 3. Maternal Comorbidity Index

eTable 4. Unadjusted Relationship Between Risk Factors and Severe Maternal Morbidity

This supplemental material has been provided by the authors to give readers additional information about their work.

© 2024 Maharjan S et al. *JAMA Network Open.*

eTable 1. ***ICD-10*** **Codes for Pregnancy Characteristics**

Condition	ICD-10 Codes
**Live birth**	Z37.0, Z37.2, Z37.50, Z37.51, Z37.52, Z37.53, Z37.54, Z37.59, Z37.3, Z37.60, Z37.61, Z37.62, Z37.63, Z37.64, Z37.69, O80
**Stillbirth**	Z37.1, Z37.4, Z37.7, O36.4XX0, O36.4XX1, O36.4XX2, O36.4XX3, O36.4XX4, O36.4XX5, O36.4XX9
**Preterm status**	O6010X0-9, O6012X0-9, O6013X0-9, O6014X0-9, O42011-9, O42111-9, O42911-9
**Full-term status**	O6020X0-9, O6022X0-9, O6023X0-9, O4202, O4292, O471, O80

© 2024 Maharjan S et al. *JAMA Network Open.*

eTable 2. Severe Maternal Morbidity Indicators

Condition	ICD-10 Codes
**1. Acute myocardial infarction**	I21.01, I21.02, I21.09, I21.11, I21.19, I21.21, I21.29, I21.3, I21.4, I21.9, I21.A1 and I21.A9, I22.0, I22.1, I22.2, I22.8, I22.9
**2. Aneurysm**	I71.00 – I71.03, I71.1, I71.2, I71.3, I71.4, I71.5, I71.6, I71.8, I71.9, I79.0
**3. Acute renal failure**	N17.0, N17.1, N17.2, N17.8, N17.9, O90.4
**4. Adult respiratory distress syndrome**	J80, J95.1, J95.2, J95.3, J95.821, J95.822, J96.00, J96.01, J96.02, J96.20, J96.21, J96.22, R09.2
**5. Amniotic fluid embolism**	O88.11x*, O88.12 (childbirth), O88.13 (puerperium)
**6. Cardiac arrest/ventricular fibrillation**	I46.2, I46.8, I46.9, I49.01[#], I49.02[@]
**7. Conversion of cardiac rhythm**	5A2204Z, 5A12012
**8. Disseminated intravascular coagulation**	D65, D68.8, D68.9, O72.3
**9. Eclampsia**	O15.00, O15.02, O15.03, O15.1, O15.2, O15.9, O14.22; O14.23
**10. Heart failure/arrest during surgery or procedure**	I97.120, I97.121, I97.130, I97.131, I97.710, I97.711
**11. Puerperal cerebrovascular disorders**	I60.0x, I60.1x, I60.2, I60.3x, I60.4, I60.5x, I60.6, I60.7, I60.8, I60.9; I61.1, I61.2, I61.3, I61.4, I61.5, I61.6, I61.8, I61.9; I62.0x, I62.1, I62.9;I63.0xx, I63.1xx, I63.2xx, I63.3xx, I63.4xx , I63.5xx, I63.6, I63.8, I63.9;I65.0x, I65.1, I65.2x, I65.8, I65.9; I66.0x, I66.1x, I66.2x, I66.3, I66.8, I66.9;I67.0, I67.1, I67.2, I67.3, I67.4, I67.5, I67.6, I67.7, I67.8xx, I67.9; I68.0, I68.2, I68.8;O22.51, O22.52, O22.53, I97.810, I97.811, I97.820, I97.821, O87.3, 674.0x
**12. Pulmonary edema and acute heart failure**	J81.0, I50.1, I50.20, I50.21, I50.23, I50.30, I50.31, I50.33, I50.40, I50.41, I50.43, I50.9
**13. Severe anesthesia complications**	O74.0; O74.1, O74.2, O74.3, O89.01, O89.09, O89.1, O89.2 O89.01
**14. Sepsis**	O85, O86.04, T80.211A, T81.4XXA, T81.44, T81.44XA, T81.44XD, T81.44XS ; R65.20, or A40.0, A40.1 , A40.3 , A40.8, A40.9, A41.01, A41.02, A41.1, A41.2, A41.3, A41.4, A41.50, A41.51, A41.52, A41.53, A41.59, A41.81, A41.89, A41.9, A32.7
**15. Shock**	O75.1, R57.0, R57.1, R57.8, R57.9, R65.21, T78.2XXA, T88.2XXA, T88.6XXA, T81.10XA, T81.11XA, T81.19XA
**16. Sickle cell disease with crisis**	D57.00, D57.01, D57.02, D57.211, D57.212, D57.219, D57.411, D57.412, D57.419, D57.811, D57.812, D57.819
**17. Air and thrombotic embolism**	I26.01, I26.02, I26.09, I26.90, I26.92, I26.99; O88.011-O88.019, 088.02, O88.03, O88.211-O88.219, O88.22, O88.23, O88.311-O88.319, O88.32, O88.33, O88.81, O88.82, O88.83 * I26.0
**18. Blood products transfusion**	99.0x à 160 ICD-10-PCS codes<br>The most common,<br>•30233H1 Transfusion of Nonautologous Whole Blood into Peripheral Vein, Percutaneous Approach<br>•30233K1 Transfusion of Nonautologous Frozen Plasma into Peripheral Vein, Percutaneous Approach<br>•30233L1 Transfusion of Nonautologous Fresh Plasma into Peripheral Vein, Percutaneous Approach

© 2024 Maharjan S et al. *JAMA Network Open.*

	•30233M1 Transfusion of Nonautologous Plasma Cryoprecipitate into Peripheral Vein, Percutaneous Approach<br>•30233N1 Transfusion of Nonautologous Red Blood Cells into Peripheral Vein, Percutaneous Approach<br>•30233P1 Transfusion of Nonautologous Frozen Red Cells into Peripheral Vein, Percutaneous Approach<br>•30233R1 Transfusion of Nonautologous Platelets into Peripheral Vein, Percutaneous Approach<br>•30233T1 Transfusion of Nonautologous Fibrinogen into Peripheral Vein, Percutaneous Approach<br>•30240H1 Transfusion of Nonautologous Whole Blood into Central vein, open approach<br>•30240K1 Transfusion of Nonautologous Frozen Plasma into Central vein, open approach<br>•30240L1 Transfusion of Nonautologous Fresh Plasma into Central vein, open approach<br>•30240M1 Transfusion of Nonautologous Plasma Cryoprecipitate into Central vein, open approach<br>•30240N1 Transfusion of Nonautologous Red Blood Cells into Central vein, open approach<br>•30240P1 Transfusion of Nonautologous Frozen Red Cells into Central vein, open approach<br>•30240R1 Transfusion of Nonautologous Platelets into Central vein, open approach<br>•30240T1 Transfusion of Nonautologous Fibrinogen into Central vein, open approach<br>•30243H1 Transfusion of Nonautologous Whole Blood into Central vein, percutaneous approach<br>•30243K1 Transfusion of Nonautologous Frozen Plasma into Central vein, percutaneous approach<br>•30243L1 Transfusion of Nonautologous Fresh Plasma into Central vein, percutaneous approach<br>•30243M1 Transfusion of Nonautologous Plasma Cryoprecipitate into Central vein, percutaneous approach<br>•30243N1 Transfusion of Nonautologous Red Blood Cells into Central vein, percutaneous approach<br>•30243P1 Transfusion of Nonautologous Frozen Red Cells into Central vein, percutaneous approach<br>•30243R1 Transfusion of Nonautologous Platelets into Central vein, percutaneous approach<br>•30243T1 Transfusion of Nonautologous Fibrinogen into Central vein, percutaneous approach<br>•30233N0 Transfusion of Autologous Red Blood Cells into Peripheral Vein, Percutaneous Approach<br>•30233P0 Transfusion of Autologous Frozen Red Cells into Peripheral Vein, Percutaneous Approach<br>•30240N0 Transfusion of Autologous Red Blood Cells into Central vein, open approach<br>•30240P0 Transfusion of Autologous Frozen Red Cells into Central vein, open approach<br>•30243N0 Transfusion of Autologous Red Blood Cells into Central vein, percutaneous approach<br>•30243P0 Transfusion of Autologous Frozen Red Cells into Central vein, percutaneous approach
**19. Hysterectomy**	0UT90ZZ, 0UT94ZZ, 0UT97ZZ, 0UT98ZZ, 0UT9FZZ

**20. Temporary tracheostomy**	0B110Z4, 0B110F4, 0B113Z4, 0B113F4, 0B114Z4, 0B114F4
**21. Ventilation**	5A1935Z, 5A1945Z, 5A1955Z

* x=1st, 2nd and 3rd trimester
[#] Ventricular fibrillation
[@] Ventricular flutter

© 2024 Maharjan S et al. *JAMA Network Open.*

eTable 3. Maternal Comorbidity Index

Condition		Weight	ICD-10 Codes
**Severe preeclampsia**		5	O14.1
**Chronic congestive heart failure**		5	I50.22, I50.23, I50.32, I50.33, I50.42, I50.43
**Congenital heart disease**		4	Q20, Q21, Q22, Q23, Q24, Q25, Q26
**Sickle cell disease**		3	D57.00, D57.01, D57.02, D57.211, D57.212, D57.219, D57.411, D57.412, D57.419, D57.811, D57.812, D57.819, (5th digit: unspecified, acute chest syndrome or splenic sequestration)
**Multiple gestation**		2	O30
**Cardiac valvular disease**		2	I05.0, I05.1, I05.2, I05.8
**Systemic lupus erythematosus**		2	M32
**Human immunodeficiency virus**		2	B20, Z21
**Mild preeclampsia or unspecified preeclampsia**		2	O14.0, O14.9
**Drug abuse**		2	F11.1, F12.1, F13.1, F14.1, F15.1, F16.1, F18.1, F19.1
**Placenta previa**		2	O44
**Chronic renal disease**		1	N26.9, N18
**Preexisting hypertension**		1	O10
**Previous cesarean birth**		1	O34.21, O34.22
**Gestational hypertension**		1	O13
**Alcohol abuse**		1	F10.1
**Asthma**		1	J45
**Preexisting diabetes mellitus**		1	O24.0, O24.1, O24.3, O24.8
**Maternal Age**			-
**35-39 years**		1	-
**40-44 years**		2	-
**45 and above**		3	-

© 2024 Maharjan S et al. *JAMA Network Open.*

eTable 4. Unadjusted Relationship Between Risk Factors and Severe Maternal Morbidity

**Characteristics**	**Adjusted OR (95% CI)**	***p-value***
**MCI**	**1.33 (1.22 - 1.45)**	**<0.001**
**Distance from delivery center**	**1.15 (1.09 - 1.21)**	**<0.001**
**Age**		
<18	0.90 (0.55 – 1.48)	0.12
18-34	1 [Reference]	
>=35	**1.86 (1.26 - 2.74)**	**0.003**
**Race**		
White	1 [Reference]	
Black	**1.40 (1.08 - 1.82)**	0.24
Others	1.17 (0.51 - 2.67)	0.97
**Pregnancy-related visit**	1.43 (1.11-1.83)	0.005
**Postpartum care visit**	1.05 (0.81 - 1.35)	0.72
**SVI**		
Least vulnerable	1 [Reference]	
Moderately vulnerable	1.02 (0.77 - 1.34)	0.60
Most vulnerable	0.91 (0.64 - 1.30)	0.51
**Level of maternity care**		
Access to maternity care	1 [Reference]	
Low access to care	0.76 (0.49 – 1.19)	0.37
Maternity care desert	0.87 (0.67 – 1.214)	0.99

Abbreviations: SVI, Social Vulnerability Index; MCI, Maternal Comorbidity Index; OR, Odds Ratio; CI, Confidence Interval

© 2024 Maharjan S et al. *JAMA Network Open.*
