# Supplementary material for: Risk Factors for Severe Maternal Morbidity Among Women Enrolled in Mississippi Medicaid
Source: JAMA Netw Open. 2024 Jan 8;7(1):e2350750. doi: 10.1001/jamanetworkopen.2023.50750 (PMC10774990; doi:10.1001/jamanetworkopen.2023.50750)
Supplement: Supplement 2. — Data Sharing Statement [file jamanetwopen-e2350750-s002.pdf]

Data Sharing Statement

Maharjan. Risk Factors for Severe Maternal Morbidity Among Women Enrolled in Mississippi Medicaid. *JAMA Netw Open*. Published January 08, 2024.
doi:10.1001/jamanetworkopen.2023.50750

Data
Data available: No

Additional Information
Explanation for why data not available: The data for this study was obtained via a data use agreement with the Mississippi Division of Medicaid, and cannot be shared publicly.
